# The R2R3 Transcription Factor CsMYB59 Regulates Polyphenol Oxidase Gene *CsPPO1* in Tea Plants (*Camellia sinensis*)

**DOI:** 10.3389/fpls.2021.739951

**Published:** 2021-11-04

**Authors:** Xiangxiang Huang, Shuqiong Ou, Qin Li, Yong Luo, Haiyan Lin, Juan Li, Mingzhi Zhu, Kunbo Wang

**Affiliations:** ^1^National Research Center of Engineering and Technology for Utilization of Botanical Functional Ingredients, Hunan Co-innovation Center for Utilization of Botanical Functional Ingredients, Hunan Agricultural University, Changsha, China; ^2^Key Laboratory of Tea Science of Ministry of Education, Hunan Agricultural University, Changsha, China; ^3^School of Chemistry Biology and Environmental Engineering, Xiangnan University, Chenzhou, China

**Keywords:** *Camellia sinensis*, polyphenol oxidase activity, transcription factor, CsMYB59, *CsPPO1* expression

## Abstract

Polyphenol oxidase (PPO) plays a role in stress response, secondary metabolism, and other physiological processes during plant growth and development, and is also a critical enzyme in black tea production. However, the regulatory mechanisms of *PPO* genes and their activity in tea plants are still unclear. In this study, we measured PPO activity in two different tea cultivars, Taoyuandaye (TYDY) and Bixiangzao (BXZ), which are commonly used to produce black tea and green tea, respectively. The expression pattern of *CsPPO1* was assessed and validated *via* transcriptomics and quantitative polymerase chain reaction in both tea varieties. In addition, we isolated and identified an R2R3-MYB transcription factor CsMYB59 that may regulate *CsPPO1* expression. CsMYB59 was found to be a nuclear protein, and its expression in tea leaves was positively correlated with *CsPPO1* expression and PPO activity. Transcriptional activity analysis showed that CsMYB59 was a transcriptional activator, and the dual-luciferase assay indicated that CsMYB59 could activate the expression of *CsPPO1* in tobacco leaves. In summary, our study demonstrates that CsMYB59 represents a transcriptional activator in tea plants and may mediate the regulation of PPO activity by activating *CsPPO1* expression. These findings provide novel insights into the regulatory mechanism of PPO gene in *Camellia sinensis*, which might help to breed tea cultivars with high PPO activity.

## Introduction

Polyphenol oxidases (PPOs) are a group of terminal copper-containing oxidases commonly found in various tissues and organs of plants, and play an important role in the enzymatic browning reaction. PPOs are mainly divided into two categories in plants: tyrosinase (EC 1.14.18.1) and catechol-oxidases (EC 1.10.3.1). Tyrosinase has monophenolase activity and catalyzes the formation of o-diphenol from monophenols, which is then oxidized to produce the corresponding quinone. Catechol-oxidases show diphenolase activity and catechol oxidase activity, which catalyze the oxidation of diphenolic compounds to generate quinone (Yu et al., 2015; Liu et al., 2019). PPOs are most abundant in young parts and less abundant in mature tissues in plants (Taranto et al., 2017). PPOs exhibit physiological functions in the growth process of plants such as resistance to stress (Mishra and Singh Sangwan, 2019), pests and diseases (Constabel et al., 2000), and regulate the formation of color in flowers (Molitor et al., 2015). In intact plant cells, PPOs are compartmentalized together with their substrates in chloroplast thylakoid membranes and vesicles, respectively (Mayer and Harel, 1979). When the cells are subjected to mechanical damage, the contact of PPO enzymes with the substrates causes rapid oxidation of polyphenols, which is responsible for the negative effects on the appearance, nutritional quality, and commercial value of agricultural products (Hojnik Podrepšek et al., 2020). However, the enzymatic browning reaction associated with PPOs is not entirely unfavorable. PPO is one of the most essential enzymes in black tea processing.

Tea is the most consumed beverage in the world and is popular worldwide due to its unique flavor and numerous health benefits (Sánchez et al., 2020; Zhu et al., 2020a,b). The fundamental difference between black tea and green tea is the occurrence of enzymatic oxidation in the former during the production process (Chen et al., 2020). The activity of PPO determines the efficiency of the withering and oxidation procedures in black tea processing (Chen et al., 2020). PPO affects a variety of tea characteristics, including color and taste, through the inhibition or enhancement of enzyme activity during tea processing. In particular, PPO and peroxidase are crucial enzymes for generating pigments during the fermentation process of black tea (Stodt et al., 2014). PPO catalyzes the oxidization of catechins in tea leaves into the orange-red theaflavins and the reddish-brown thearubigins, as well as catalyze the enzyme-coupled oxidation of fragrance precursors (Solano et al., 1997). Theaflavins and thearubigins are the major polyphenols that determine the quality of black tea. Theaflavins contribute to the color, astringency and the brightness of black tea liquor, and thearubigins are positively correlated aftertaste of astringency in black tea (Owuor et al., 2010; Wang et al., 2014). Therefore, PPO activity plays a key role in the development of black tea characteristics.

Currently, the PPO gene family in *Camellia sinensis* is thought to contain five to six members (Zhao et al., 2001). Zhang et al. (2020) find that methyl jasmonate enhances PPO activity by activating the expression of *CsPPO2* and *CsPPO4* gene to improve defense against tea geometrid larvae (*Ectropis grisescens*) in tea plant. Additionally, mechanical damage or regurgitant of tea geometrid significantly upregulates the transcriptional expression of *CsPPO1* and *CsPPO2* (Huang et al., 2018). These findings indicate that increasing the expression of PPO genes in tea plants is of interest; however, the molecular mechanism of the regulation of PPO activity in tea plants remains unclear.

Transcription factors (TFs) have been shown to increase gene expression in various systems. TFs represent a specific group of proteins that regulate stress and signaling in plants, and bind to *cis*-acting elements in the promoters of downstream genes to regulate gene expression (Riechmann et al., 2000; Amoutzias et al., 2007). MYB proteins are a TF superfamily with diverse functions. Based on the number of incomplete repeats within the MYB conserved domain, MYBs are classified into four categories (1R-, R2R3-, R3-, and 4R-) (An et al., 2018). The R2R3-MYB subfamily is the largest class of MYB TFs in plants, regulating a variety of physiological functions at the transcriptional level, including plant growth and development, metabolite synthesis, and resistance to stress (Stracke et al., 2001). Currently, 126, 109, and 140 R2R3-MYB members have been identified in *Arabidopsis thaliana* (Dubos et al., 2010), *Oryza sativa L.* (Feller et al., 2011), and *C. sinensis* (Chen et al., 2021), respectively. In tea plants, R2R3-MYBs are involved in regulating the biosynthesis of flavonoids and theanine (Wang et al., 2018; Wen et al., 2020). In the purple foliage mutant phenotype, the R2R3-MYB TF CsAN1 regulates the expression of anthocyanin biosynthesis-related genes causing ectopic accumulation of pigments in purple tea (Sun et al., 2016). In addition, the *Arabidopsis-*derived AtMYB96 TF can modulate the expression of abscisic acid and auxin-related genes to regulate drought-related stress response (Seo et al., 2009). Overexpression of MYB49 in tomato significantly enhances the resistance of tomato plants to *Phytophthora infestans*, and increases the superoxide dismutase activity, peroxidase activity, chlorophyll content, and photosynthetic rate (Cui et al., 2018). Recently, the R1R2R3 TF MnMYB3R1 derived from mulberry plants (*Morus notabilis*) has been shown to upregulate PPO by binding to MSA in the promoter region of *MnPPO1* (Liu et al., 2019). However, no study has reported on whether TFs can regulate PPO activity in tea plants.

In this study, *C. sinensis* var. *sinensis* cv. Taoyuandaye (TYDY) with high PPO activity and superior fermentation characteristics & *C. sinensis* var. *sinensis* cv. Bixiangzao (BXZ) with low PPO activity and inferior fermentation characteristics were used. Analysis of transcriptomic data was performed between TYDY and BXZ. The expression patterns of *CsPPO1* and R2R2-MYB TF *CsMYB59* were analyzed *via* quantitative real time polymerase chain reaction (qRT-PCR). These results improve the current understanding of the regulatory mechanisms of PPO gene in tea leaves, which provide theoretical support for the further innovation of high-quality tea cultivar.

## Materials and Methods

### Tea Plant Materials

Leaves were obtained from clonal tea plants cultivated in Tea Germplasm Repository of Hunan Agricultural University, Changsha, Hunan, China. The leaves of one bud with two leaves of similar growth and development without mechanical damage were picked, flash frozen using liquid nitrogen, and stored at −80°C.

### Determination of Polyphenol Oxidase Activity in Tea Leaves

Polyphenol oxidase activity was measured using the spectrophotometric method (Teng et al., 2017) with modifications. In brief, 0.5 g fresh leaves were homogenized with 2 g quartz sands and 0.3 g polyvinylpyrrolidone (Sigma-Aldrich, Shanghai, China) in 4.5 mL, pH 5.6, 100 mM cooled citric acid-phosphate buffer (citric acid and phosphate were purchased from Sinopharm chemical reagent, Shanghai, China), and the volume was set to 10 mL by adding the buffer following extraction at 4°C for 12 h. After filtration, the homogenate was centrifuged at 8000 × *g* for 15 min at 4°C and the supernatant containing the crude enzyme was collected. Crude enzyme (0.2 mL) was added to a mixture of 100 mM catechol (0.5 mL, Sinopharm chemical reagent, Shanghai, China) and 100 mM citric acid-phosphate buffer (1 mL), and then the absorbance of the resultant mixture was measured at 420 nm for 20 min. One unit (U) of PPO activity was expressed as 0.01 U in Δ420/min, and each sample were measured thrice.

### RNA Extraction, Sequencing, and cDNA Synthesis

Total RNA was extracted from tea leaves using an RNA extraction kit (Tiangen, Beijing, China). Total RNA quality and concentration were assessed *via* agarose gel electrophoresis and RNA Nano 6000 Assay Kit (Agilent Technologies, Santa Clara, CA, United States), respectively. A cDNA library was synthesized using a reverse transcription kit (Takara, Shiga, Japan); duplicates of each sample were measured thrice. The cDNA library was quantified using a Qubit 2.0 fluorometer (Invitrogen, Waltham, MA, United States), and an Agilent 2100 Bioanalyzer (Agilent Technologies) was used for quality assessment. The cDNA library was sequenced using Illumina HiSeqTM 2000 (Illumina, San Diego, CA, United States). Clean reads were filtered from the raw reads by deleting reads containing adaptors and low-quality reads. The *C. sinensis* reference genome was downloaded from https://pcsb.ahau.edu.cn:8080/CSS/ (Wei et al., 2018). Transcripts were aligned to the reference tea plant genome using Hisat2 v2.0.5 (Kim et al., 2015). Gene expression levels were normalized by calculating fragments per kilobase of transcript per million mapped reads (FPKM).

### Gene Cloning and Expression Analysis

The upregulated MYB gene *CsMYB59* was selected and identified from the TYDY transcriptome (unpublished) and *C. sinensis* genome (Wei et al., 2018), and aligned to the reference *C. sinensis* genome. The full-length sequence of *CsMYB59* was amplified with specific primers which were designed according to the reference genome (primers are shown in Supplementary Table 1). *CsMYB59* was cloned using 2X PRO Taq Master Mix kit (Accurate Biology, Changsha, China) under the following parameters: pre-denaturation at 94°C for 30 s, subsequent denaturation at 98°C for 10 s, annealing at 58°C for 10 s, and elongation at 72°C for 1 min. Amplification was performed in 35 cycles. Finally, the PCR cloning was completed at 72°C for 5 min. The full-length sequence of *CsMYB59* (750 bp) was obtained after cloning and sequencing (Supplementary Table 2). Gene expression analysis was carried out by qRT-PCR with a SYBR Green Premix Pro Taq HS qPCR Kit (Accurate, Hunan, China) on an ABI QuantStudio 3 Real-Time PCR system (Thermo Fisher, United States). The reaction parameters of qRT-PCR were described as our previous published study (Luo et al., 2018).

### Gene Identification and Characterization Analysis

Amino acid sequences were translated using the National Centre for Biotechnology Information (NCBI) ORF finder^1^. The physical and chemical characteristics of the protein were predicted using ProParam tool^2^. Homology analyses of protein sequences were performed using NCBI basic local alignment search tool (BLAST) database^3^. The multiple alignment of amino acid sequences was performed using the MUSCLE program in MEGA X (Kumar et al., 2018), and then phylogenetic trees were generated *via* the Neighbor--Joining method with a bootstrap number 1000. The NCBI Conserved Domain Database (CDD^4^ was used to identify the domains of the protein sequences that were edited using Genedoc software (Nicholas et al., 1997).

### Promoter Isolation and Analysis

DNA was extracted from tea leaves using the cetyl trimethylammonium bromide method. The promoter sequence of the *CsPPO1* gene (TEA026892.1) was obtained from the reference tea genome^5^. Full length *CsPPO1* promoter amplification primers (Supplementary Table 1) were designed using Premier 5.0. The *CsPPO1* promoter was isolated *via* PCR. After sequencing, the promoter sequence of *CsPPO1* with a length of 1016 bp was obtained. The Plant Care online software^6^ was used to predict the *cis*-acting elements of *CsPPO1* promoter.

### Subcellular Localization of CsMYB59 in Tobacco (*Nicotiana benthamiana*) Plants

Subcellular localization was predicted using the Plant-Ploc^7^ online software. To confirm the subcellular localization prediction, the *CsMYB59* coding sequence without the stop codon was cloned into a pEAQ-green fluorescence protein (GFP) vector (Sainsbury et al., 2009) to generate the fusion protein CsMYB59-GFP. Subsequently, both the pEAQ-CsMYB59-GFP and pEAQ-GFP vectors (Sainsbury et al., 2009) were transformed into *Agrobacterium tumefaciens* strain EHA105 (Weidi Biotechnology Co., Ltd, Shanghai, China), and the constructs were injected into tobacco leaves (Yu et al., 2018). After incubation for 2 days, the green fluorescence signals were analyzed using an LSM 710 laser-scanning confocal microscope (Carl Zeiss, Oberkochen, Germany) (Wen et al., 2020).

### Transcriptional Activation in Yeast and Tobacco

Transcriptional activity assays were performed using a yeast system. The coding sequence of *CsMYB59* was cloned into a pGBKT7 vector (Clontech, Mountain View, CA, United States) to generate a BD-CsMYB59 expression vector. The BD-CsMYB59 vector and a control BD empty vector were transformed into *Saccharomyces cerevisiae* Y2H Gold (Weidi Biotechnology Co., Ltd, Shanghai, China). The yeast cells were cultivated in synthetic defined (SD) medium (Sangon Biotech Co., Ltd., Shanghai, China) lacking tryptophan (SD/–Trp) at 28°C for 48–96 h. The transformed yeast cells were then cultured in SD medium without tryptophan, histidine, and adenine (SD/-Trp/-His-Ade) at 28°C for 48–72 h. Transcriptional activity of *CsMYB59* was determined using X-α-galactosidase (Sangon Biotech Co., Ltd., Shanghai, China). In addition, the full-length sequence of the CsMYB59 TF was cloned into a pBD vector to generate pBD-CsMYB59 as an effector. The double-reporter vector was driven by 35S promoter, and contained GAL4-LUC and an internal control *Renilla* luciferase (REN). The GAL4-LUC contains 5X GAL4, minimal TATA region, and the *Luciferase* (LUC) (Vo et al., 2017). Mixtures of the effector and reporter were injected into tobacco leaves. After incubation for 2 days, The LUC/REN ratio in tobacco leaves was determined using a Dual Luciferase Reporter Assay Kit (Vazyme Biotech, Nanjing, China) as described by Wen et al. (2020) to confirm the transcriptional activity of *CsMYB59* (Luo et al., 2018).

### Dual-Luciferase Reporter Assay

To investigate the transcriptional effect of the CsMYB59 TF on the promoter of *CsPPO1*, the CsMYB59-pEAQ vector was used as an effector, whereas a pGreen II 0800-LUC vector containing the promoter fragments of *CsPPO1* was used as a reporter. The mixtures of effector and reporter were injected into tobacco leaves. After incubation for 2 days, The LUC/REN ratio in tobacco leaves was determined to evaluate the transcriptional effect of CsMYB59 on the *CsPPO1* promoter gene as described by Luo et al. (2018).

### Statistical Analysis

All the experiments were performed in triplicate and all data are presented as the mean ± standard deviation. Statistical analysis was performed using Student’s *t*-tests. A value of *P* < 0.05 or *P* < 0.01 was considered significant.

## Results

### Analysis of Polyphenol Oxidase Enzyme Activity in Leaves of Different Tea Plant Cultivars

**FIGURE 1:**
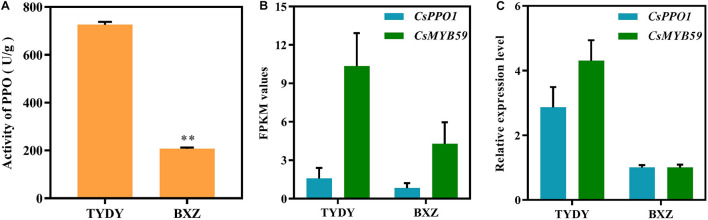
Polyphenol oxidase activity and gene expression of *CsPPO1* and *CsMYB59* in different tea cultivar leaves. **(A)** PPO activity in TYDY and BXZ. **(B)** The FPKM values of *CsPPO1* and *CsMYB59* in TYDY and BXZ. **(C)** The relative expression level of *CsPPO1* and *CsMYB59* in TYDY and BXZ. PPO, polyphenol oxidase; TYDY, Taoyuandaye; BXZ, Bixiangzao; FPKM, fragments per kilobase of transcript per million mapped reads. **represents significant difference at *P* < 0.01.

To investigate the difference in PPO activity between TYDY and BXZ, spectrophotometric analysis was performed to determine the PPO activity. The PPO activity in TYDY was significantly higher than that in BXZ (Figure 1A; *P* < 0.01).

### Sequencing and Transcriptome Mapping

Six cDNA libraries of TYDY and BXZ (three biological replicates for each cultivar) were generated for transcriptome sequencing. Based on the reference *C. sinensis* transcriptome assembly (Wei et al., 2018), a total of 339 million reads were identified after raw-read quality filtering, with an average of 56.6 million clean reads in each library. The average Q30 and GC content were 91.96 and 44%, respectively. Reference-based mapping of quality reads yielded 89.18% (TYDY) and 89.01% (BXZ). In addition, the uniquely mapped reads were 83.79–84.43% (Supplementary Table 3).

### Analysis of the Expression Pattern of *CsMYB59* and *CsPPO1*

The PPO activity in tea leaves is regulated by the expression of *CsPPO* genes. To investigate the regulatory mechanism of CsMYB59 TF in the effect on PPO activity in tea leaves, we measured the FPKM values of *CsMYB59* and *CsPPO1* genes *via* RNA sequencing (RNA-seq). By analyzing the differences in PPO activity between the two tea cultivars, we found that the expression level of *CsPPO1* was positively correlated with PPO activity (Figures 1A,B). In addition, the FPKM value in TYDY was higher than that in BXZ which was consistent with the trend of *CsPPO1* expression.

The gene expression levels of *CsPPO1* and *CsMYB59* were analyzed *via* qRT-PCR. The expression level trends of *CsPPO1* and *CsMYB59* genes were consistent with the transcriptomic data (Figures 1B,C). These results indicated that the CsMYB59 TF may have a positive correlation with *CsPPO1* expression and PPO activity.

### Identification and Bioinformatics Analysis of *CsMYB59*

**FIGURE 2:**
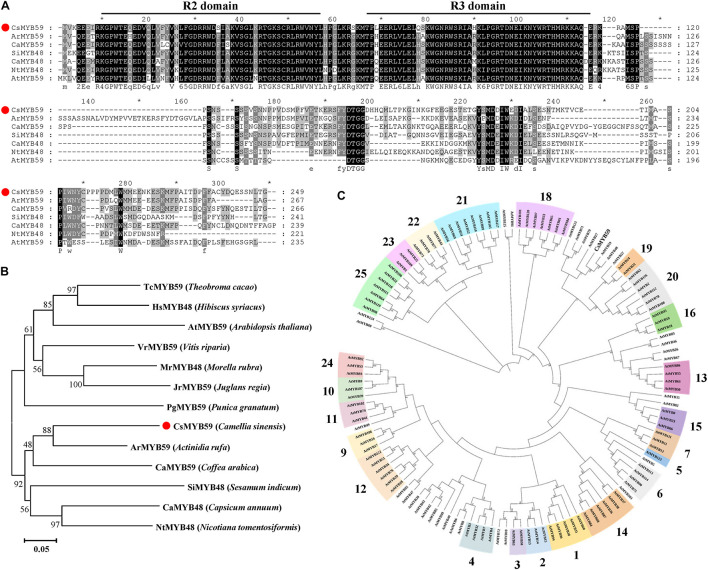
Bioinformatics analysis of the CsMYB59 protein sequence. **(A)** Protein sequence alignment between CsMYB59 and ArMYB59, CaMYB59, SiMYB48, CaMYB48, NtMYB48, and AtMYB59. **(B)** Phylogenetic tree generated using sequences of CsMYB59 and homologous MYB transcription factors of 12 other plant species; CsMYB59 is labeled with a red dot. The plants are specified in the brackets. The accession numbers of the protein sequences are as follows: TcMYB59 (EOY34142.1), HsMYB48 (XP_038991988.1), AtMYB59 (Q4JL84.2), VrMYB59 (XP_034687869.1), MrMYB48 (KAB1226039.1), JrMYB59 (XP_035545886.1), PgMYB59 (XP_031387608.1), ArMYB59 (GFS42876.1), CaMYB59 (XP_027108891.1), SiMYB48 (XP_011089426.1), CaMYB48 (KAF3662000.1), and NtMYB48 (XP_009590049.1). **(C)** Phylogenetic analysis of CsMYB59 and *Arabidopsis*-derived R2R3-MYB sequence. The bold numerals represent subgroups of R2R3-MYB TFs in *A. thaliana*. *means positions that have a single, fully conserved residue. CsMYB59 is marked with a red dot.

The *CsMYB59* sequence cloned from TYDY was analyzed using the ExPASy software for physicochemical properties (Supplementary Table 4). The open reading frame of *CsMYB59* was 750 bp and encoded a protein containing 249 amino acids with a predicted molecular mass of 28.93 kDa. The theoretical isoelectric point was 6.55, the instability index was 76.57, and the average hydrophilic coefficient was −0.859. These data indicated that CsMYB59 may be an unstable hydrophilic protein. Analysis using the NCBI CDD database revealed that CsMYB59 was similar to other homologous MYB TFs and contained a conserved R2R3-MYB DNA-binding domain at the N-terminus (Figure 2A), which may perform analogous functions.

To further evaluate the molecular properties of CsMYB59, phylogenetic trees were generated. Homology analysis was performed using the amino acid sequences of CsMYB59 and MYB proteins from 12 other plant species (Figure 2B). Moreover, a neighbor-joining analysis was performed using the CsMYB59 and *A. thaliana* R2R3-MYB amino acid sequences (Figure 2C). Results showed that CsMYB59 was homologous to *Actinidia rufa-*derived ArMYB59 (64.23%). MYB59 has been reported to play a central role in biotic and abiotic stress responses in plants (Hickman et al., 2017; Fasani et al., 2019). *AtMYB48* and *AtMYB59* are homologous genes involved in the response to nutrient deprivation in *Arabidopsis* (Nishida et al., 2017). In addition, AtMYB59 participates in modulating calcium homeostasis and signal transduction induced by excessive cadmium levels (Fasani et al., 2021). These findings indicate that CsMYB59 may be involved in the response to stress and secondary metabolite biosynthesis in tea plants.

### Analysis of Subcellular Localization of CsMYB59

**FIGURE 3:**
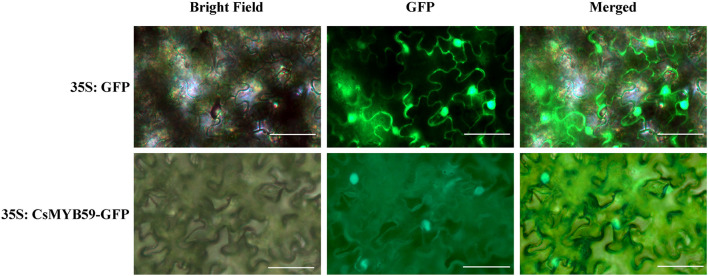
Subcellular localization analysis of CsMYB59 protein. The GFP- and CsMYB59-GFP-fused proteins were transfected into the epidermal cells in *Nicotiana benthamiana* leaves. The white bars represent 50 μm. GFP, green fluorescent protein.

Subcellular localization analysis predicted that CsMYB59 was localized in the nucleus and may be a nuclear protein. Subcellular localization assays using tobacco plants verified this result (Figure 3). The control GFP fluorescence signal was observed in tobacco leaf epidermal cells, whereas the CsMYB59-GFP signal was detected only in the nuclear region. This result indicated that CsMYB59 may be a nuclear protein showing transcriptional activity.

### Transcriptional Activity Analysis of CsMYB59

**FIGURE 4:**
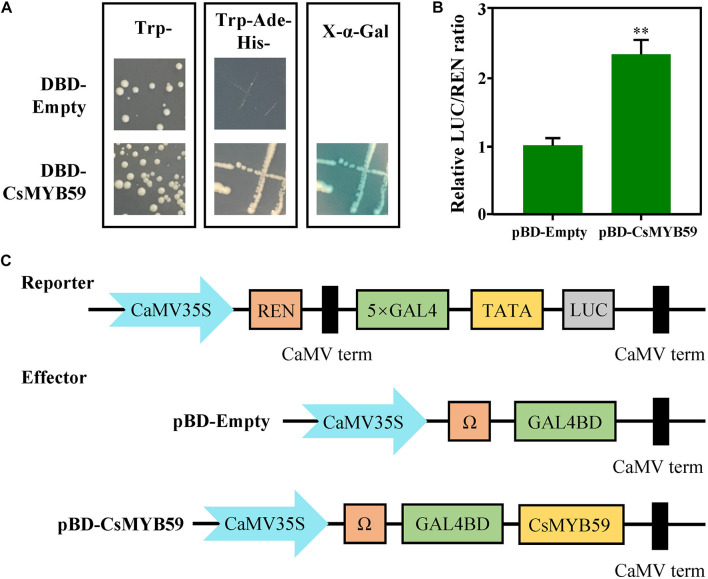
Transcriptional activity analysis of CsMYB59. **(A)** Transcriptional activation of CsMYB59 in *Saccharomyces cerevisiae*. *CsMYB59* was subcloned into a pGBKT7 vector and then transformed into yeast cells. The transformed yeast cells were grown on selective SD mediums (SD/-Trp/-His/-Ade), and further selected *via* X-Gal assays. **(B,C)** The relative REN/LUC ratio of CsMYB59 and pBD-empty was calculated to evaluate the transcriptional activity of CsMYB59. Transcriptional activation of CsMYB59 in tobacco. The transcriptional activation of CsMYB59 was verified *via* dual-luciferase reporter assays using *Nicotiana benthamiana* leaves. ^∗∗^ represents significant difference at *P* < 0.01. SD, synthetic defined; -Trp/-His/-Ade, media lacking tryptophan, histidine, and adenine; LUC, luciferase; REN, *Renilla* luciferase; X-Gal, X-α-galactosidase.

To investigate the transcriptional activity of CsMYB59, we used a GAL4-responsive reporter system in yeast. The transformed yeast carrying CsMYB59-BD showed growth when cultivated in SD/-Trp/-His/-Ade selective medium and showed α-galactosidase activity which indicated that CsMYB59 activates transcription as an activator (Figure 4A). In addition, the transcriptional activity of CsMYB59 was evaluated *via* dual-luciferase assays in *N. benthamiana* leaves. Compared to the pBD-empty vector, the ratio of LUC/REN in the pBD-CsMYB59 was significantly higher than that in the control (Figures 4B,C). Therefore, the role of CsMYB59 as a transcriptional activator was verified.

### CsMYB59 TF Regulated the Expression of *CsPPO1* Promoter

**FIGURE 5:**
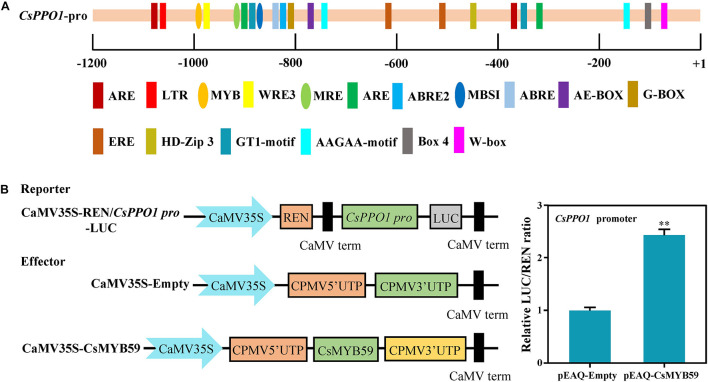
**(A)** *Cis* element analysis of *CsPPO1* promoter gene. The axis represents the cloned *CsPPO1* promoter sequence. Ellipses represent elements that can bind to MYB transcription factors. Rectangles represent other elements. **(B)** Ability of CsMYB59 to activate transcription of *CsPPO1* promoter in *Nicotiana benthamiana*. The relative REN/LUC ratio of pEAQ-CsMYB59 and pEAQ-empty was calculated to evaluate the transcriptional regulatory effects of CsMYB59 on *CsPPO1* promoter. ^∗∗^ represents significant difference at *P* < 0.01. LUC, luciferase; REN, *Renilla* luciferase.

Analysis of the *cis*-acting elements (Figure 5A) showed that the promoter region of *CsPPO1* contained several specific MYB-binding *cis*-elements such as MYB (5′-CAACCA-3′), MBSI (5′-TTTTTACGGTTA-3′), and MER (5′-AACCTAA-3′) (Smita et al., 2015). To further investigate the transcriptional regulatory effects of CsMYB59 on *CsPPO1*, dual-luciferase assays were performed using *N. benthamiana* leaves. The constructed CsMYB59-pEAQ vector was used as the effector, and *CsPPO1 pro*-LUC was used as the reporter. Compared to the control which was transformed with an empty vector, the co-transformation of CsMYB59 and *CsPPO1 pro*-LUC significantly increased the ratio of LUC/REN (Figure 5B). This result suggested that CsMYB59 may activate the expression of *CsPPO1*, and may be positively correlated with PPO activity.

## Discussion

Polyphenol oxidase exhibits multiple important physiological effects such as resistance to stress, pests and diseases during growth and development (Huang et al., 2018). During the manufacturing processes of black tea, the flavonoids present in the leaves are oxidized to o-quinones by PPO that are associated with the formation of pigments such as thearubigins, theaflavins, and theabrownin. The oxidized catechins can promote the accumulation of aroma compounds in tea by oxidizing amino acids, carotenoids, fatty acids, and other substances (Shi et al., 2014). Therefore, PPO activity is of great importance with regard to the quality improvement of black tea. The PPO gene family members are typically encoded by small gene families. Seven members of the PPO family have been identified in tomato plants (Kowalski et al., 1992), six members have been identified in the family of potato plants (Thipyapong and Steffens, 1997), and five to six members have been identified in *C. sinensis* (Zhao et al., 2001). PPOs of different gene family members contain highly conserved copper-binding domains; however, the gene expression of PPOs is specific and functionally diverse. The *PPO1* gene in barley is a major determinant in regulation of the phenol reaction in awns (Taketa et al., 2010). *Cis*-acting elements that interact with MYB TFs have been detected in the *SmePPO1* gene in eggplants, which regulates various metabolic reactions (Shetty et al., 2012). Domain analysis of *CsPPO1* and *CsPPO2* in *C. sinensis* showed that *CsPPO1* and *CsPPO2* both contain Tyrosinase, PPO1_DWL, and PPO1_KFDV domains. Among them, *CsPPO1* contain two tyrosinase domains, Tyrosinase_1 and Tyrosinase_2, whereas *CsPPO2* contains only Tyrosinase_2 (Supplementary Table 5; Cary et al., 1992). *CsPPO1* may play various roles due to the presence of two Tyrosinase domains. In this study, the PPO activity of two different tea cultivars was analyzed, and we found that the PPO activity in TYDY was significantly higher than that in BXZ. Furthermore, the *CsPPO1* gene expression was significantly higher in TYDY than in BXZ based on the RNA-seq data, and this result was consistent with the trend of PPO activity; qRT-PCR analysis verified this result. Therefore, increasing the expression of *CsPPO1* may positively regulate PPO activity in tea plants.

TF CsMYB59, whose expression follows the same pattern of variation in PPO activity among different tea cultivars, was isolated based on the transcriptomic data. Bioinformatics analysis indicated that CsMYB59 belonged to the R2R3-MYB TF family (Figure 2A). In *C. sinensis*, 122 R2R3-CsMYB genes have been identified and classified into 25 subgroups; CsMYB59 belongs to the S24 subgroup (Chen et al., 2021). Phylogenetic analysis showed that CsMYB59 was highly homologous to ArMYB59 (64.23%, *A. rufa*) compared to MYB TF sequences from other plants, suggesting that these proteins may have similar functions. It is well known that the R2R3-MYB TF families are involved in plant growth and development (Stracke et al., 2007), stress response (Wong et al., 2016), and primary and secondary metabolism (Blanco et al., 2018). Nishida et al. (2017) found that two homologous genes, AtMYB48 and AtMYB59, show altered DNA-binding sites created by alternative splicing in response to environmental stresses of potassium deficiency. AtMYB59 is involved in the response to *Heterodera schachtii* infestation in *A. thaliana* (Wisniewska et al., 2021). In addition, an R2R3 TF ZmMYB31 isolated from *Zea mays* can enhance superoxide dismutase and ascorbate peroxidase activities to protect plants against low temperature stress (Li et al., 2019). These findings suggest that R2R3-MYB TFs play an important role in plant metabolite synthesis and resistance to stress. Due to the fact that one of the main functions of PPO is involved in resistance to stress in tea plant, the CsMYB59 may has a regulatory role on PPO activity.

*Cis*-acting element analysis revealed that *CsPPO1* contained multiple MYB-binding sites such as G-BOX and MRE which are involved in the light response (Hartmann et al., 1998, 2005), MBSI which is involved in the regulation of flavonoid biosynthetic genes (Zhang et al., 2016), and ERE and MYB of unknown functions. These binding sites may control downstream gene expression by binding MYB TFs. Recently, an R1R2R3 TF MnMYB3R1 is isolated from *Morus notabilis* which upregulates PPO by binding to MSA in the promoter region of *MnPPO1* (Liu et al., 2019). Therefore, it is important to increase the PPO activity by regulating TFs so as to cultivate high stress resistant tea cultivars and improve the quality of black tea. However, there are no studies on tea plant-derived TFs regulating PPO gene expression. Our results suggest that an R2R3-MYB TF *CsMYB59* expression may be positively correlated with the gene expression of *CsPPO1*. In addition, qRT-PCR verified the expression pattern of *CsMYB59* in association with *CsPPO1*; the transcriptomic data were consistent with the qRT-PCR data. Based on these results, we speculate that CsMYB59 may regulate the *CsPPO1* promoter.

In total, 98.36% of R2R3-CsMYB TFs are localized in the nucleus (Chen et al., 2021). Similarly, our study showed that CsMYB59 may represent a nuclear protein. MYB TFs have been reported to operate as transcriptional activators as well as transcriptional repressors. NtMYB3 inhibits flavonol biosynthesis in *Narcissus* by suppressing its FLS promoter activity (Anwar et al., 2019). CsMYB73 in *C. sinensis* binds to the promoter regions of *CsGS1* and *CsGS2 via* MYB recognition sequences and represses the transcriptional activity of *CsGS1* and *CsGS2*, thereby participating in the biosynthesis of_L_-theanine in tea plants (Wen et al., 2020). AtMYB2 activates *miR399f* expression and regulates the Pi-starvation responses in *Arabidopsis* by operating as a transcriptional activator that binds directly to the MYB-binding site in the *miR399f* precursor promoter (Baek et al., 2013). In the present study, we found that CsMYB59 showed transcriptional activity in yeast cells, and the results were verified *via* transcriptional activity analysis in tobacco leaves which confirmed that CsMYB59 may function as a transcriptional activator. Furthermore, dual-luciferase assays indicated that CsMYB59 activated the transcription of the promoter of *CsPPO1*, and may regulate PPO activity in tea plants by activating *CsPPO1*.

Currently, several studies suggest that MYB interacts with transcription factors such as bHLH and WD40 to regulate the expression of promoters (Grotewold et al., 2000; Zimmermann et al., 2004). For example, Liang et al. (2014) showed that AtMYB82 can interact with GL3, and is integrated into the WD40-bHLH-MYB complex to participate in *Arabidopsis* trichome development. Tea plant-derived CsWD40 interacts with two bHLH TFs (CsGL3 and CsTT8) and two MYB TFs (CsAN2 and CsMYB5e) to form a ternary WBM complex that regulates anthocyanin and pro-anthocyanidin biosynthesis, and trichome development (Liu et al., 2018). In this study, we found that the *CsPPO1* promoter contains a large number of elements that can bind to other transcription factors, such as W-Box. Therefore, further studies on the regulatory relationships between CsMYB59 and other *CsPPOs*, and the interactions of other transcription factors may help us further understand the regulatory mechanisms of PPO in tea plants, and will provide a theoretical basis for the screening and breeding of premium tea plant germplasm resources.

## Conclusion

In this study, we evaluated the PPO activity in two different tea cultivars, TYDY and BXZ, and isolated and identified a TF, CsMYB59, that may exert regulatory effects on PPO activity in tea plants. CsMYB59 is a transcriptional activator that may specifically activate the expression of *CsPPO1*, thereby positively regulating PPO activity. These findings expand the knowledge of the transcriptional regulation mechanism of R2R3-MYB TFs involved in PPO metabolism in tea plants and provide potential theoretical supports for the molecular breeding and innovation of tea cultivar resources with high PPO activity.

## Data Availability Statement

The original contributions presented in the study are included in the article/Supplementary Material, further inquiries can be directed to the corresponding author/s.

## Author Contributions

KW and YL designed the research. SO carried out experiments. XH processed the data and wrote the manuscript. MZ, HL, QL, and JL revised the manuscript. All authors approved the manuscript.

## Conflict of Interest

The authors declare that the research was conducted in the absence of any commercial or financial relationships that could be construed as a potential conflict of interest.

## Publisher’s Note

All claims expressed in this article are solely those of the authors and do not necessarily represent those of their affiliated organizations, or those of the publisher, the editors and the reviewers. Any product that may be evaluated in this article, or claim that may be made by its manufacturer, is not guaranteed or endorsed by the publisher.

## Footnotes

1
https://www.ncbi.nlm.nih.gov/orffinder/

2
https://web.expasy.org/protparam/

3
http://www.ncbi.nih.gov/BLAST/

4
https://www.ncbi.nlm.nih.gov/Structure/cdd/wrpsb.cgi

5
https://pcsb.ahau.edu.cn:8080/CSS/

6
http://bioinformatics.psb.ugent.be/webtools/plantcare/html/

7
http://www.csbio.sjtu.edu.cn/bioinf/plant/
