# Supplementary material for: The R2R3 Transcription Factor CsMYB59 Regulates Polyphenol Oxidase Gene CsPPO1 in Tea Plants (Camellia sinensis)
Source: Front Plant Sci. 2021 Nov 4;12:739951. doi: 10.3389/fpls.2021.739951 (PMC8600361; doi:10.3389/fpls.2021.739951)
Supplement: Supplementary file 1 [file Data_Sheet_1.doc]

Table S1. List of primers used in this study

| Prime names | Prime sequence (5'-3') | Usage |
| --- | --- | --- |
| CsMYB59 | F：ATGGTGAAAGAGGAGGAGATCAGG | Full-length gene cloning |
|  | R：TTAGCCGGTTAAATTCGAGCTCTCTTG |  |
| CsPPO1 | F：TTGGCACAAAGCAGAAGGTTGTC |  |
|  | R：ATGGTGGCGGCGGAGGCCGCGGTGG |  |
| CsMYB59 | F：GGGGAGGACCGACAAT | qRT-PCR |
|  | R：GGCGGGAAACATCTTACT |  |
| CsPPO1 | F：AATCCCGACACTCCGAAACC |  |
|  | R：GCAGCACTGTAACCGCCTAAC |  |
| GAPDH | F：TTGGCATCGTTGAGGGTCT |  |
|  | R：CAGTGGGAACACGGAAAGC |  |
| GFP-CsMYB59 | F：caaattcgcgaccggt ATGGTGAAAGAGGAGGAGATCAGG | Subcellular localization |
|  | R：tgctagtcataccggt GCCGGTTAAATTCGAGCTCTCTTG |  |
| BD-CsMYB59 | F：catggaggccgaattc ATGGTGAAAGAGGAGGAGATCAGG | Transcriptional activity |
|  | R：gccgctgcaggtcgacg TTAGCCGGTTAAATTCGAGCTCTCTTG |  |
| pBD-CsMYB59 | F：tcgccgaccggtaggcct ATGGTGAAAGAGGAGGAGATCAGG | transient expression |
|  | R：aaccagagttaaaggcct GCCGGTTAAATTCGAGCTCTCTTG |  |
| pEAQ-CsMYB59 | F：caaattcgcgaccggt ATGGTGAAAGAGGAGGAGATCAGG |  |
|  | R：agttaaaggcctcgag GCCGGTTAAATTCGAGCTCTCTTG |  |
| 0800-CsPPO1 | F：tatagggcgaattgg GTACCTTGGCACAAAGCAGAAGGTT |  |
|  | R：ttggcgtcttccatgg GGGTTTGGGTATTGGGGTGTA |  |

Table S2. Full-length gene sequence of *CsPPO1* and *CsMYB59*

| Gene | Full-length gene sequence |
| --- | --- |
| *CsPPO1* | TTGGCACAAAGCAGAAGGTTGTCTTTGAGGAATGAATTACATAGCAGCCCTTGCAAATTATATGATAATAAGTTGAAGTACTTGACCAAAAATGGATAACATTAATTTCACATTACACATTAAAAAAAACTACTTAATAAAATTTGGTAAAGCAAGCCACATTTATTGACAAAATCAACAAAGATGACTTCTTTTAAAAGAGAACCATTTCATTTTGCCTTGGCAGAGGAATTACTAGCCATATTATCAATTTTATTTGTATACAAATCCAATAGCCTTAAAATTAGATATCCTAAATTGAGATCATAAAGGGTTGGTTTGGGAAGTGGGTTAGCTTTTTGGCTTTT..TAGTTTTTTTGGCTATTTTAGCCTAGGTTAAAAAGTTTGGTTTGGGAAGAACTTTTTGACTTTTTGACTTTTTTGACTTTTTTTATGAGAGAATATTGTAATGAT.TATGGGTTGGATGTGATGTTTTGTTTGTTGTGACTAAAATAATATATTTTGTCTTTTATGACTAAAATAGTCAAAAAAGCCCACAAAACACTTCCCAAACGGTGCAATATATATTAAAATTCTCAAAATTTTAAAAAATTTGTTCAAAAAGTACTCAGAATTTTAAAATTATAGAACAAATTTATGTTAGACAAACTCTACATACTATATATATTAAAATTCTCAACATTTTTTAAAAAAATAAAAATTCTGATTTACATACCATTTCTTTAAACGCAATCAGACAAAAACTAGCAACCCAAGAAAGAAGAAAAAGTTAAATTAAATCCAAATCAAGTTTCTACGACTTGGGAAGTCATTTTTTTCCACCACCACGTGGTGTTTGTAGTTTTTTATTTGTCAACTTGAACAACCCAGCTGTCTGTGTCATTACGTAGAAAAGTCTTAACCCTAAAAACCATTATATAAGCATAGCTAATAACCTAATGGCTACACCCCAATACCCAAACCCATGGCTTCTTTTCCACCTTCAACCACCACCACCGCGGCCTCCGCCGCCACCATCACCCCAACTTCTTCGCCATTTTTCAAAAAAACCCTTCAAATCCCGACACTCCGAAACCA |
| *CsMYB59* | ATGGTGAAAGAGGAGGAGATCAGGAAGGGACCATGGACAGAACAAGAAGATGTACAACTGGTTTTCTATGTGAATTTGTTTGGTGATCGGAGATGGGACTCCATAGCAAAAGTTTCAGGCTTGAAAAGAACAGGAAAGAGTTGCAGGTTGCGTTGGGTCAATTACCTCCACCCGGGTCTCAAAAGGTCCAAGATGACTCCCCTTGAAGAACGCCTTGTCTTGGAACTTCAGTCCAAATGGGGAAATAGATGGTCAAGAATTGCTCACAAGTTGCCGGGGAGGACCGACAATGAGATCAAGAACTACTGGAGGACTCACATGAGGAAGAAGGCTCAGGAGAGGAAAAGGGCCATGTCACCACCTTCAAATTCATCTTCCTCGGTCTCAAACAATCCTCCGGTGGACTCGATGCCCTTCGTGGAGACCAAGGAACGAAGCTTCTACGATACCGGTGGGGATCATCATCAGATGTTGACTCCAAAGGGAATAAATAAGGGTTTTGAAGAGGGAGAGAGTACCGAAACAGGGTACTCCATGGATGACATATGGGAAGACATTGCTCTATCTGAAAGCAACACGATGAAAACAGTTTGTGAAACTATCATGGCTTCTCCAATTTGGAACTACTGTCCTCCTCCTCCTGACATGCTTTGGATGATGGAAGAAAATAAGGAGAGTAAGATGTTTCCCGCCATAACCGATCCATTTTTTGCATGTTATGATCAAGAGAGCTCGAATTTAACCGGCTAA |

Table S3. Summary of the TYDY and BXZ transcriptome (*Camellia sinensis*)

| Sample name | Raw reads | Clean reads | Total mapped (%) | Uniquely mapped (%) | Clean bases | Error rate (%) | Q20 (%) | Q30 (%) | GC content (%) |
| --- | --- | --- | --- | --- | --- | --- | --- | --- | --- |
| TYDY 1 | 59083418 | 58591488 | 89.4 | 84.43 | 8.79 G | 0.03 | 97.09 | 92.12 | 44.74 |
| TYDY 2 | 58864774 | 58203592 | 89.48 | 84.55 | 8.73 G | 0.03 | 97.30 | 92.56 | 44.65 |
| TYDY 3 | 61689148 | 61028528 | 88.65 | 83.79 | 9.15 G | 0.03 | 97.15 | 92.27 | 44.46 |
| BXZ 1 | 54543438 | 54003872 | 88.9 | 83.96 | 8.1 G | 0.03 | 96.73 | 91.36 | 44.44 |
| BXZ 2 | 54064298 | 53547852 | 89.15 | 84.26 | 8.03 G | 0.03 | 96.80 | 91.46 | 44.53 |
| BXZ 3 | 54528736 | 54004412 | 88.97 | 84.08 | 8.1 G | 0.03 | 97.25 | 92.49 | 44.94 |

Table S4. The physicochemical properties of CsMYB59 proteins

|  | Number of amino acids (aa) | Molecular weight (Da) | Electronic point (PI) | Instability index | Aliphatic index | Grand average of hydropathicity |
| --- | --- | --- | --- | --- | --- | --- |
| Max | 1365 | 152126.31 | 10.55 | 85.09 | 90.81 | -0.185 |
| Min | 146 | 16790.36 | 4.75 | 31.16 | 54.21 | -1.051 |
| Ave | 384 | 42846.72 | 7.132 | 53.11 | 70.37 | -0.663 |

Table S5. The prediction domains analysis of CsPPOs amino acid sequences

| Gene name | Start | Stop | Location | Mode | Sequence | |
| --- | --- | --- | --- | --- | --- | --- |
| *CsPPO1* | 202 | 219 | TYROSINASE_1 | H-x(4,5)-F-[LIVMFTP]-x-[FW]-H-R-x(2)-[LVMT]-x(3)-E | Hnswl.FFpFHRyyLhffE | |
|  | 360 | 371 | TYROSINASE_2 | D-P-x-F-[LIVMFYW]-x(2)-H-x(3)-D | DPiFFghHaniD |  |
| *CsPPO2* | 350 | 361 | TYROSINASE_2 | D-P-x-F-[LIVMFYW]-x(2)-H-x(3)-D | DPmFFahHgnvD | |
